# Observations and Experiments on Mesmerism

**Published:** 1829-06

**Authors:** Richard Chenevix


### MESMERISM.

*Observations and Experiments on Mesmerism.* By RICHARD CHENEVIX, Esq. F.R. and E.S. M.R.I.A.

It is by no means the desire of those who are convinced of the truth of mesmerism to urge belief upon their mere assertion, but to excite curiosity; to turn the public mind toward this powerful agent, so true, yet so much despised; and to engage some inquirers to lay aside their preconceptions for a moment, and have recourse to fair experiment. The difficulty of overcoming the first disbelief, the first repugnance to actual trial, is extreme; but, as the authority of some respectable medical men, whom their own senses and their own experience have induced to practise this art, may contribute to remove it, the following cases are submitted to the public:

"*Ballynacarig (Ireland); February* 5*th*, 1829.

"Dear sir,—In the month of September last, a few days after I had first witnessed the wonderful effects of mesmerism in your hands, I happened to pay a professional visit at Mears court, in this neighbourhood. While there, I learned that a man, named Hubert Gainer, was at the point of death.

"I should inform you that this man, for the last four or five years, had been continually under my care, in the dispensary, for very bad dyspepsia, particularly marked by the most obstinate state of the bowels, requiring generally a double quantity of the strongest purgatives every three or four days.

"On arriving at his house, I found him stretched out in bed, with the stomach inordinately distended, and his thirst excessive. His extremities were cold, his countenance ghastly, his pulse small and quick, his tongue white; his bowels confined for the last five days. He was writhing in torture from pain in his stomach. I learnt, on inquiry, that he had had no rest for the two preceding nights.

"Though at that time I was far from believing in the practical utility of mesmerism, yet finding that in the case of this patient something should be done immediately, I proceeded to try my hand at mesmerism, in imitation of what I had seen you do. After some time the man grew quiet; when, with the hope of exciting the stomach to action, I directed my intention particularly to the epigastric region. Continuing this for two or three minutes, the man turned round suddenly, and, to my surprise, vomited an immense quantity of liquid, (the most acrid bile.) He then threw himself back into a profound sleep, which continued for an hour and a half. At this period I gradually awoke him, when he was evidently better in every respect. Leaving directions that his wife should report his case to me in the dispensary next morning, I took my departure. By the account I received the following day, I found that he had slept soundly, and that his bowels had been freed three times in the night: in short, his wife reported next morning that he was perfectly well. In a few days I called to see him, and found him complaining of weakness only; but his bowels were again confined, and again I had recourse to mesmerism, with the same good effect.

" I resorted to mesmerism in this man's case but three times in all, and he is, as I have seen this day, in perfect health; neither have I had occasion to give him any aperient medicines since the first application of this surprising influence.

" As the above case appears, to me at least, highly interesting, I take the liberty of giving it to you somewhat in detail; and have the honour to be, dear sir,

" Your most obedient servant,

" R. COTTER, M.D."

" To Richard Chenevix, Esq. Sonna."

Michael Donally had been attended for some time by Dr. Cotter, who pronounced him to be far advanced in rapid consumption. He was taking small but repeated doses of tartar emetic and digitalis. On February 11th, I mesmerised him for the first time. He was in bed, and exceedingly weak; his voice was hardly audible. The only sensible effect produced during the operation was profuse perspiration.

Feb. 12th.—After I had left him yesterday, he slept for about an hour, and, on waking, found his cough and breathing easier. He had left off the tartar emetic and digitalis, as well as all other medicines. Mesmerised him thirty minutes, with the same effect as yesterday.

Feb. 13.—Found him going on very well. His voice was stronger, and he seemed more alive than I had yet seen him.

As I could not visit him every day, I instructed his wife how to proceed and desired her to mesmerise him night and morning, during thirty minutes each time. I also informed Dr. Cotter that I had undertaken this desperate case, adding a hope that we still might save the patient. Dr. Cotter's reply was, "If the poor man is saved, I will substitute the pronoun *you* for *we.*"

Daily accounts were brought to me of the progress of this man until February 27th, when I called upon him, and found him up and dressed. He received me at the door of his cottage, spoke with a strong, firm voice, looked healthy, and said he was nearly recovered. I told his wife to persevere in the mesmeric treatment.

March 16th.—He came to see me, and looked quite well. I mesmerised him for a few minutes: he slept, and even showed some interesting phenomena. I urged his wife to continue the treatment some time longer; for mesmerism, when persevered in after the cure is effected, is never dangerous.—This case can be attested by at least twenty witnesses of the first respectability.

The two following cases were communicated to me by Mr. Levinge, of the Royal College of Surgeons, Dublin, with permission to publish them:

February 9th, 1829.—Mary Spotten, æt. sixteen, had for several months been complaining of a severe and continual pain in her back, with a fixed and numb sensation shooting from the bottom of the abdomen down the inside of the thighs. She was much emaciated; her countenance was pale and yellow; her appetite quite lost. When I first prescribed for her, about six months ago, she was essentially relieved; and in a short time the regular discharge appeared, but in small quantities.

"After this time I did not see her for two months, when I learnt from her mother that she was as ill as ever. A considerable period then elapsed, during which nothing was attempted for her. I had, in the mean time, heard much of the salutary effects produced by mesmerism under the care of Mr. Chenevix, and, through the kindness of this gentleman, had seen two or three of his patients who had been cured by him, and many others who had been considerably relieved. Instructed by him respecting the mode of applying the mesmeric influence in these charitable performances, my opinion, which had hitherto been strongly prejudiced against the doctrine, became so changed, that, on returning home, I sent for this girl, and commenced to try mesmerism upon her. The operation was repeated daily at the same hour, and continued for thirty minutes each time. The first days she did not sleep, but said she felt as if her heart had ceased to beat. After a few repetitions of the operation, however, sleep came on regularly, and usually continued while that lasted. Now and then, on awaking, she complained of vague pains in her head, belly, and legs; but they immediately ceased when I directed my attention to those parts. During this time she took not one particle of medicine, but every day she drank a quart of mesmerised water. At the end of one month she was discharged cured; but the treatment appears not to have been continued long enough, for, during a suspension of it, necessarily caused by my absence, her illness has returned.

"Biddy Connell, æt. eleven, had for some time been afflicted with cough, loss of appetite, and slight bronchitis, and had passed several small worms. About three months since, she applied to me for relief. Every day, for three weeks, I performed the operation of mesmerism upon her during thirty minutes. After the first four days she passed many worms, and felt much better;* her cough began also to diminish gradually. At the end of a fortnight, the quantity of worms which she had voided was very great, and the relief which she experienced was as remarkable. She also drank magnetised water. After the treatment had been continued for three weeks, I was obliged to go to Dublin; but the girl was recovered; neither has she had any return of her illness since that time.

"This patient did not sleep more than two minutes at a time; and, as soon as she opened her eyes, she seemed as completely awake as if she had not slept at all."

The following is extracted from an intended publication, now preparing for the press, and in which the results of experiments and observations made upon 442 patients will be detailed. Ten months were devoted to these laborious but interesting investigations, and six, eight, sometimes ten hours a day, were allotted to their prosecution uninterruptedly.

A common opinion among persons who admit the truth of mesmerism, but who are little acquainted with its practice or effects, is that it is most efficacious upon nervous temperaments. Having tried this agency pretty extensively upon other disorders, and having been led to an opposite conclusion, I was anxious to put it to the proof in a disease which may be considered as the very epitome of nervousness, insanity. With great eagerness I embraced an opportunity offered to me by the kindness of Mr. Higgins, whose name, so well known among the benefactors to that wretched class of beings whom malady has deprived of reason, deserves to be remembered as long as humanity exists among men. Informed of the power of mesmerism, and satisfied as to its general truth, Mr. H. proposed to me to try it in his neighbourhood, where I then happened to be,

* I have myself cured seven cases of worms.

in the lunatic asylum of Wakefield, in Yorkshire; an establishment where madness ceases almost to be a misfortune, which, in any other country of Europe, would be extolled as one of the greatest of national monuments, but which in England rears its immense but modest bulk in a sequestered vale, and is hardly heard of beyond the district where it spreads its blessings. Thither Mr. Higgins had the goodness to accompany me, and there, under the auspices of Dr. Ellis, physician to the establishment, the experiments were made. The subjects selected for trial were two males and eight females. One of the males was furious and manacled. The first ten minutes produced no sensible effect upon him; but afterwards his head twice sunk down upon his chest, with an evident tendency to somnolency, which, however, was soon disturbed by his suddenly starting up almost as frantic as before. Two women, afflicted with melancholy, were tried, but with no sensible effect: neither was the success more apparent upon any of the remaining subjects, two only excepted.

A woman, whose reason was much less subverted than that of the other patients, said that every time I drew my hand before her, she "felt life going down through her body along with them," to use her own words; a feeling analogous to that which many delicate persons have described.

A girl, aged eighteen, epileptic as well as insane, showed symptoms of somnolency in one minute after the operation began; and during half an hour, which it lasted, was three times in mesmeric sleep. But she always started suddenly out of that state, into which she again fell in one minute. One general effect, however, struck all the attendants who accompanied these patients in the room where the experiments were made. A state of calmness was produced as soon as the passes began, and continued during the whole operation. Even the furious man became more sedate while under the action of mesmerism; and a girl, who never could be prevailed upon to remain quiet, required less coercion from her attendant than in ordinary circumstances. The trial upon each patient never exceeded thirty minutes; and these experiments can by no means be considered as conclusive respecting the therapeutic effects of mesmerism upon insanity. Neither were they intended to be so. The object was to compare the immediate physiological influence of this agent upon persons in a healthy and in a deranged state of mind: and even in this point of view they are of very limited value. As far, however, as one trial, of half an hour each, upon ten insane persons, can authorize an inference, it must be concluded that the influence is considerably less upon them than upon persons of sound mind; and still less again than upon persons inflicted with other infirmities. Two medical gentlemen attached to the establishment, together with Dr. and Mrs. Ellis, were present at these trials; and, if the reality of mesmerism had hung upon the results, the science would be in a sad plight. The effects were so weak as hardly to be perceptible to persons wholly unacquainted with mesmeric phenomena, whose inexperience well might question their existence, and whose preconceptions would allow them to discern nothing but failure in all that occurred. Neither was conviction expected to follow these trials, or any other consequence but a comparison between persons sane and insane. But truth, though often clouded, will burst forth; and a little triumph was reserved for that which is the great guide of man, in a world where the utmost light he can attain is just enough to make darkness visible.

In the evening's conversation, Dr. Ellis mentioned an extraordinary complaint to which he was subject in his stomach. I requested to mesmerise him for fifteen minutes. At the expiration of two minutes his countenance became flushed, his eyes a little bloodshot, and he smiled; he soon afterwards closed his eyes. When the fifteen minutes were elapsed, seeing that he was not asleep, I asked him how he found himself? "I never felt so comfortable in my life. I feel as if I should like to remain in this state always, and never more to move hand or foot." "Pray let me ask you," said I, "why you smiled at the end of two minutes?" "I smiled to find all my incredulity oozing out of me." "You do, then, from your own feelings, acknowledge an effect in mesmerism?" "I do; I must." Dyspeptic cases are among those in which I have found the curative influence of mesmerism the most prompt and efficacious.

It must not be concluded from the above that mesmerism is ineffectual upon insanity. In the "Exposé des Cures operées en France par le Magnitisme Animal," two vols. a work indispensable to all who study this science, eight cures are instanced as effected by a continued treatment. Among sixty-seven epileptic patients whom I myself have treated, two were afflicted with mental derangement, and many with temporary alienation, for several hours after each epileptic paroxysm. In no one instance did the latter symptom fail of being alleviated. In the two insane subjects, the intensity of both diseases was diminished; but, though one patient was magnetised fifty-four times, and the other seventy-six, neither was entirely cured. As I left the country where they resided, the continuation of the treatment was confided, with proper instructions, to their relations.

From the very extraordinary power which mesmerism possesses in other diseases, it is much to be hoped that it may be tried in insanity also; and that no prejudice, no sneers of wilful ignorance or of inconvincible presumption, will longer oppose the application of a means, which, in every other country in Europe, has been practised with miraculous success; which reckons thousands of converts in Germany, Switzerland, Italy, Holland, and France; and which has been admitted by Hufeland, Jussieu, Cuvier, Ampere, and Laplace.

The preceding extract was submitted to Dr. Ellis for his sanction, and the following answer was returned:

"*Wakefield Asylum; April* 9, 1829.

"Dear sir,—I have carefully read your extract, and find it perfectly true and candid. For myself, I have no hesitation in declaring that I felt very sensibly affected by the operation; for, where truth and science are to be promoted, no fear of being laughed at shall ever deter me from avowing my sentiments. I have no doubt, too, that I should have fallen asleep if we had been alone; but, surrounded as we were by so many spectators, the very consciousness of feeling the sensation coming upon me, as you truly observe, made me smile, and aroused me. I am too ignorant upon the subject of mesmerism to say any thing upon it that is worth your attention; but, on reflecting upon it, it has struck me that there must be a great difference in the susceptibility of persons being affected by it, according to their states of health, temperaments, &c. All circumstances relating to it should be carefully noted down; and, as soon as you have put the matter into a tangible shape, I shall certainly give it my most serious and candid attention."

Since the experiments were made at Wakefield, I have repeated similar trials in some ordinary hospitals, in the presence of several spectators; and the results obtained have induced me to modify my opinion respecting the limited success which attended them in that institution. The state which mesmerism tends to produce is a state of quietude and calmness. Numerous witnesses disturb and annoy both the patient and the operator. The latter, too, becomes uneasy at seeing his patient agitated, and his anxiety takes away from him the power of concentrating his mind; a condition far more necessary to produce effects than any mental state of the person acted upon. It is then possible that the spectators, whose presence was an annoyance to Dr. Ellis, may have disturbed the insane patients; and that insanity may be as susceptible of mesmeric influence as any other disorder. We are at present, with regard to mesmeric knowledge, nearly in the same state as was the first man who saw a straw attracted by a piece of amber, with respect to electricity.

Three medical practitioners, two M.D.'s and one surgeon, were thus convinced of the truth of mesmerism by experiments made either by them or on them: and experiment is the test to which the proselytes of this doctrine demand that it should be put. Their first cry is for experiment; their second for experiment; their third for experiment.

The ignorance of the medical world in this country upon mesmerism is as great as the precipitancy with which the question is prejudged. Every work upon it current either in Germany or France, has been slighted and despised. The accounts published at Berlin of the cures performed in the Mesmeric Hospital there, since 1815, have not been listened to. The extraordinary case of Mademoiselle Samson, witnessed successively by thirty-two physicians of the Faculty of Paris, at the Hôtel Dieu, is unknown here, though it has been the subject of so much discussion there. The still more wonderful cure of Paul at the Charité, though attested by near two hundred credible witnesses, has met only with contempt from the very few medical men in England who have ever heard of it. The deliberations of the French Academy of Medicine, in which nearly one half of its members confess that they have seen and believe mesmeric phenomena most marvellous and important, have never been laid before the British public as they should have been. The common opinion is that mesmerism still is what it was forty-five years ago, and that it was then laid to rest for ever by the report of Bailly, Darat, Lavoisier, Franklin, &c. This article shall be concluded with an extract from that report, and with the more recent opinions of two men, whose authority even sceptics must respect. The words of the report are as follows:

"The patients (submitted to mesmeric experiments,) present a very varied picture of results. Some are calm, quiet, and perceive no effect; others cough, spit, feel slight pains, partial or general heat, and perspire; others are tormented and agitated by convulsions: those convulsions are extraordinary for their force and duration. As soon as one convulsion begins, others declare themselves, and your commissioners have seen some which lasted more than three hours. Nothing can be more astonishing than the sight of these convulsions. He who has not beheld them can form no idea of them; and, even in beholding them, one is equally surprised at the profound repose in which some of the patients are plunged, and at the agitation which animates others. It is impossible not to recognize in these effects, *which are constant*, a great power which agitates the patients, which ever masters them, and of which *the person who magnetizes seems to be the depositary*. This convulsive state is improperly denominated *crisis** in the theory of animal magnetism."

This is the report which the present opposers of mesmerism invoke, as having given an eternal quietus to the science, and in which they say that the phenomena are denied. Yet even this report, far as it is from not admitting mesmeric facts, and from countenancing the interpretation which is often given of it, as great a man as any who signed it, Jussieu, thought unworthy of the subject, and made a contradictory report. Jussieu,—aut deus, aut Jussieu,—well accustomed to interrogate nature, instituted separate experiments, which fully proved the reality of mesmerism, and authorized the conclusion that imagination plays as great a part in mesmeric phenomena as in any other physiological phenomena, and *no greater*. The fact is, that ninety-nine experiments in a hundred refute the hypothesis of imagination.

Let M. Cuvier now be heard. In the 117th page of the second volume of his Comparative Anatomy he says, "It must be confessed that in all experiments, the object of which is to determine the action which the nervous system of one person may have upon the nervous system of another, it is difficult to distinguish the effects of the imagination of the person acted upon, from the physical effects produced by the person who acts. Yet the effects produced upon persons who, before the operation was begun, were in a state of insensibility; those which have taken place upon other persons, after the operation itself had reduced them to that state; and also the effects produced upon animals, no longer permit it to be doubted that the proximity of two animated bodies, in a certain position, and with the help of certain motions, do produce a *real effect*,

* The modes and the results of mesmerism have been almost as much modified since 1784 as those of electricity since the days of Pliny, and convulsions now are unusual phenomena. Of 442 patients whom I have mesmerised in ten months, and sixty-seven of whom were epileptic or hysterical, but eight had convulsions. These belonged to the latter sixty-seven, and the paroxysms were very speedily relieved.

*wholly independent of the imagination of either.* It is also evident that these effects are entirely owing to a communication which takes place between the nervous system of the two parties."

The last evidence which shall now be heard is LAPLACE. The words (translated) of this great man, in his "Traité analytique du Calcul des Probabilités," page 158, are, "The extraordinary phenomena which result from the extreme sensibility of the nervous system in some persons, have given birth to a variety of opinions on the existence of a new agent, denominated animal magnetism. It is natural to suppose that the influence of these causes is very weak, and that it can easily be disturbed by accidental circumstances; but it would be unfair to conclude that it never exists, merely because in many cases it does not manifest itself. We are so far from being acquainted with all the agencies of nature, and with their different modes of action, that it would be unphilosophical to deny the existence of phenomena solely because, in the present state of our knowledge, they are inexplicable to us." I have myself had more than one conversation with M. Laplace upon this subject, about the years 1816 and 1817, and his expression constantly was, that the testimony in favor of the truth of mesmerism, coming with such uniformity from enlightened men of many nations, who had no interest to deceive, and possessed no possible means of collusion, was such that, applying to it his own principles and formulas respecting human evidence, he could not withhold his assent to what was so strongly supported.

Now, though the superiority of British intellect has no more strenuous advocate than myself, I must say that it would not disgrace the greatest man whom England ever has produced to attempt an experiment or two upon a doctrine which Hufeland, Jussieu, Cuvier, Ampere, and Laplace believed. Nay, would it not disgrace him more to contemn, without knowing any thing about it, what they knew and credited? Is supercilious ignorance the weapon with which Bacon would have repelled a new branch of knowledge, however extraordinary it might have appeared to him? and would not Newton have repeated the experiments of Euler and of Dollond, before he would have dared to deny them? Surely, what great men believe ordinary men may try. Then, if they err, they will have noble companions, with whom it may be an honour to go astray: if they are right, they will stand with mighty minds and truth upon their side.

If mesmerism be wilful deception and juggling, this is the country whose duty it is to expose the imposture. If it be venial error and delusion, England, the chief supreme of intellectual judicature, should rectify it. If it be true, she should not be the last to acknowledge it. But, mesmerisers come forward under the broad ægis of experiment. By experiment let the truth be told. Let any twelve men in England devote twelve half hours each to experiment, *secundum artem*, and then relate the issue.

The books which give the best account of the present state of mesmerism are, "Histoire critique du Magnetisme Animal," by M. Deleuze; "Instructions pratiques sur le Magnetisme Animal," by the same; "Traité du Somnambulism," by Bertrand; "Exposées des Cures operées en France par le Magnetisme Animal depuis Mesmer jusqu'a nos jours," 2 vols.; "Annales du Magnetism;" "Bibliotheque Magnetique;" "L'Hermes," and "Le Propagateur," two periodical journals, exclusively destined to mesmerism. The fifteen volumes of cases published in Berlin, and in German, are the richest collection of mesmeric transactions existing. The works of Hufeland, Kieser, Wolfart, &c. are also of the highest value.

See vol. 62, p. 114.

